# STRUCTURAL BASES OF GASTROINTESTINAL MOTILITY CHANGES IN PARKINSON’S DISEASE: STUDY IN RATS

**DOI:** 10.1590/0102-672020200003e1548

**Published:** 2021-01-15

**Authors:** José Cirlanio Sousa ALBUQUERQUE, Tiago Santos MENDES, Maria Girlane Sousa Albuquerque BRANDÃO, Annyta Fernandes FROTA, Thomas Dominik de Souza dos REIS, Lissiana Magna Vasconcelos AGUIAR, José Ronaldo Vasconcelos da GRAÇA

**Affiliations:** 1 Physiology and Neuroscience Laboratory at the Biotechnology Graduate Program at the Medical School, Sobral, CE, Brazil; 2 UNINTA University center, Sobral, CE, Brazil; 3 Nursing Graduate Program, Luso Afro-Brazilian University, Campus in Redenção, CE, Brazil; Department of Biochemistry and Molecular Biology, Federal University of Ceará, Pici Campus, Fortaleza, CE, Brazil

**Keywords:** Neurodegeneration, 6-Hydroxidopamine, Pathophysiology, Constipation, Vagotomy, Neurodegeneração, 6-Hidroxidopamina, Fisiopatologia, Constipação, Vagotomia

## Abstract

**Background:**

Gastrointestinal disorders are frequently reported in patients with Parkinson’s disease whose disorders reduce the absorption of nutrients and drugs, worsening the clinical condition of patients. However, the mechanisms involved in modifying gastrointestinal pathophysiology have not yet been fully explained.

**Aim:**

To evaluate its effects on gastrointestinal motility and the involvement of the vagal and splanchnic pathways.

**Methods:**

Male Wistar rats (250-300 g, n = 84) were used and divided into two groups. Group I (6-OHDA) received an intrastriatal injection of 6-hydroxydopamine (21 µg/animal). Group II (control) received a saline solution (NaCl, 0.9%) under the same conditions. The study of gastric emptying, intestinal transit, gastric compliance and operations (vagotomy and splanchnotomy) were performed 14 days after inducing neurodegeneration. Test meal (phenol red 5% glucose) was used to assess the rate of gastric emptying and intestinal transit.

**Results:**

Parkinson’s disease delayed gastric emptying and intestinal transit at all time periods studied; however, changes in gastric compliance were not observed. The delay in gastric emptying was reversed by pretreatment with vagotomy and splanchnotomy+celiac gangliectomy, thus suggesting the involvement of such pathways in the observed motor disorders.

**Conclusion:**

Parkinson’s disease compromises gastric emptying, as well as intestinal transit, but does not alter gastric compliance. The delay in gastric emptying was reversed by truncal vagotomy, splanchnotomy and celiac ganglionectomy, suggesting the involvement of such pathways in delaying gastric emptying.

## RESUMO

**Racional:**

Distúrbios gastrintestinais são frequentemente relatados em pacientes com doença de Parkinson cujos distúrbios reduzem a absorção de nutrientes e fármacos, agravando o quadro clínico dos pacientes. No entanto, os mecanismos envolvidos na alteração da fisiopatologia gastrintestinal ainda não foram totalmente elucidados.

**Objetivo:**

Avaliar os seus efeitos sobre a motilidade gastrintestinal e o envolvimento das vias vagal e esplâncnica.

**Métodos:**

Ratos Wistar machos (250-300 g, n=84) foram utilizados e divididos em dois grupos. O grupo I (6-OHDA) recebeu injeção intraestriatal de 6-hidroxidopamina (21 µg/animal). O grupo II (controle) recebeu solução salina (NaCl, 0,9%) nas mesmas condições. O estudo do esvaziamento gástrico, trânsito intestinal, complacência gástrica e operações (vagotomia e esplancnotomia) foram realizadas 14 dias após a indução da neurodegeneração. Refeição teste (vermelho de fenol+glicose 5%) foi utilizada para avaliar a taxa de esvaziamento gástrico e o trânsito intestinal.

**Resultados:**

A doença de Parkinson retardou o esvaziamento gástrico e o trânsito intestinal em todos os tempos estudados; porém, alterações da complacência gástrica não foram observadas. O retardo do esvaziamento gástrico foi revertido por pré-tratamento com vagotomia e esplancnotomia+gangliectomia celíaca, sugerindo assim, o envolvimento de tais vias nos distúrbios motores observados.

**Conclusão:**

A doença de Parkinson compromete o esvaziamento gástrico, bem como o trânsito intestinal, mas não altera a complacência gástrica. O retardo do esvaziamento gástrico foi revertido pela vagotomia troncular, esplancnotomia e gangliectomia celíaca, sugerindo o envolvimento de tais vias no retardo do esvaziamento gástrico.

## INTRODUCTION

Parkinson’s disease (PD) is the second most prevalent neurodegenerative disease in the world, affecting 1% of the population over 55 years old^16^. Estimates demonstrate that it could affect more than 10 million worldwide by 2030^10^. Its main neuropathological characteristic is the lesion of dopaminergic neurons that reduce the levels of dopamine in the striatum, in addition to the appearance of Lewy bodies*,* formed by the accumulation of α-synuclein and ubiquitin proteins^7^ ^,^ ^2^. Damage to dopaminergic neurons dramatically affects different regions of the brain, midbrain, brain stem, olfactory tubercle, cerebral cortex and elements of the peripheral nervous system^3^. 

The main symptoms of PD are: tremors, stiffness and bradykinesia; however, others appear during the progression of the disease, such as postural instability, autonomic dysfunction, cognitive deficits, psychiatric disorders, sensory losses and sleep disorders^6^. Gastrointestinal disorders - constipation, difficulty in chewing, delayed gastric emptying, dry mouth or excessive salivation, dysphagia and gastroesophageal reflux - are reported in approximately 70% of patients^4^. Reduction in the rate of gastrointestinal emptying affects the absorption of levodopa, causing fluctuations in motor symptoms, reducing the quality of life for patients^8^. In addition, recent clinical trials have shown that 40-60% of people with gastroparesis also overgrow bacteria in the small intestine^11^.

 Gastrointestinal motility is controlled by coordinated mechanisms in both the central and enteric nervous systems^2^ ^,^ ^11^. Studies conducted with animal models suggest that delayed gastrointestinal emptying may be associated with an impairment of the vagal and splanchnic pathways. However, the pathophysiology mechanisms of the brain-intestinal axis in PD have still not been completely explained^14^.

Among various experimental models, the neurodegeneration induced by unilateral injection of 6-hydroxydopamine (6-OHDA) in animals is one of the most used, capable of providing important information to clarify the understanding regarding the pathophysiology of the disease, enabling the development of new therapeutic strategies^5^. 

Due to the abovementioned, the objective of this study was to evaluate the effects of PD on gastrointestinal motility and the involvement of vagal and splanchnic pathways on rats.

## METHODS

### Animals and ethical aspects

Male Wistar rats (250-300 g, n=84) from the central vivarium of the Federal University of Ceará were kept in a room with controlled environmental conditions (25±1° C, humidity 60±5%, 12 h light/dark cycle) with free access to water and food (Nuvital Nuvilab rat food - CR1-Nuvital Nutrientes S/A, Brazil). All experiments were carried out according to the Ethical Principles Guide for the Care and Use of Laboratory Animals, proposed by the Brazilian Society of Laboratory Animal Science after approval by the local ethics committee (protocol N° 40/2015). All efforts to minimize the number and suffering of the animals were implemented.

### Neurodegeneration induction

The animals were initially divided into two groups (n=6), they received with ketamine (100 mg/kg, i.p) and xylazine (5 mg/kg, i.p) for anesthesia purposes, followed by a stereotaxic surgery. Group I (6-OHDA) received a unilateral intrastriatal injection of 6-OHDA (20 µg/animal) on the ipsilateral side. Group II (control) received a saline solution (NaCl, 0.9%) under the same conditions. The unilateral intrastriatal injection of 6-OHDA or the saline solution was performed using a Hamilton^®^ 6 μL syringe and a stereotactic device (Stoelting, USA) with the following coordinates (mm): site 1: L: - 2.5, AP: + 0.5, V: + 5.0; Location 2: L: - 3, AP: - 0.5, V: + 6.0; and location 3: L: - 3.7, AP: - 0.9, V: + 6.5 of bregma, according to the Paxinos and Watson Atlas^1^. 

### Rotational behavior assessment

The rotational behavior was evaluated by monitoring the rotations induced by apomorphine (3 mg/kg i.p) which induces the animal to rotate in the opposite direction of the injury (contralateral side). The number of rotations (360°) around the axis was counted every 10 min for a total period of 60 min^17^.

### Gastric emptying assessment

The gastric emptying study was performed 14 days after the neurodegeneration induction operation. After 12 h of fasting, the animals were fed (via gavage) with 1.5 ml of liquid test meal containing a non-absorbable marker (0.5 mg/ml of phenol red in 5% glucose solution). After 10, 20 or 30 min, the rats were sacrificed by cervical dislocation and the intestine was exposed by laparotomy, fixed to the pylorus, cardia and ileocecal junction, and then removed. The intestine was carefully stretched from the stomach to the colon and divided into four consecutive segments: stomach, proximal small intestine (first 40%), medial small intestine (intermediate 30%) and distal small intestine (last 30%). Each segment was placed in graduated cylinders containing 100 ml of NaOH 0.1 N. After being crushed and homogenized for 30 s, the sediment suspension was left for 20 min at room temperature. The supernatant (10 ml) was centrifuged for 10 min at 2800 rpm and the proteins contained in 5 ml of the homogenate volume were precipitated after adding 0.5 ml of trichloroacetic acid (20% w/v). After centrifugation for 20 min at 2800 rpm, 3 ml of the supernatant was added to 4 ml of 0.5 N NaOH. A standard dilution curve was generated to relate the concentration of phenol red in 0.1 N NaOH with the absorbance (560 nm) obtained in each segment^9^ ^,^ ^15^. The fractional recovery of gastric dye was determined according to the following equation: % of gastric recovery = amount of phenol red recovered in the stomach/total amount of phenol red recovered from all four segments × 100.

### Intestinal transit assessment

After anesthesia with ketamine (100 mg/kg, i.p) and xylazine (5 mg/kg, i.p), the animals were submitted to laparotomy and a cannula was inserted into the intestine. Its tip was moved to the duodenum 1 cm distal to the pylorus and fixed to the stomach wall; its free end was channeled subcutaneously, externalized and attached to the skin. After two days, the animals were fed (gavage) with the liquid test meal (1 ml) through the duodenal cannula and were sacrificed 20 min later. After exeresis, the intestine was carefully stretched and removed. Obstructive bandages were placed to obtain five consecutive segments of the small intestine (~20 cm long). Each segment was homogenized, and the dye content was determined by spectrophotometry. The data obtained for each individual segment was multiplied by the total number of segments and added to calculate the geometric center of the marker distribution throughout the intestine^9^ ^,^ ^15^.

### Assessment of gastric compliance

To assess the effects of PD on gastric compliance, a barostat system associated with a plethysmometer was used. After 14 days of the neurodegeneration induction operation, the animals were anesthetized with urethane (1.2 g/kg i.p) followed by a tracheostomy. A balloon catheter (~4 ml) made with the fingertips of surgical gloves was inserted orally and positioned in the stomach of the rats. The free end was connected to a glass reservoir (2.5 cm internal diameter, 30 ml volume), creating a system of communicating vessels filled with a standard ionic solution (45 mg% NaCl and 0.3 ml% of Imbebiente, BBC Ornano, Comerio, Italy) at 37° C. Thus, changes in the volume of the reservoir, displayed by the plethysmometer (model 7140, Ugo Basille, Comerio, Italy) were considered gastric volume. After the system was balanced, the stomach was progressively distended, increasing the liquid level in the reservoir 4, 8 and 12 cm above the rat’s xiphoid appendix every 10 min. Gastric volume was recorded every 1 min and collected at consecutive 10 min intervals^13^.

### Neuroautonomic pathways involvement study

The animals initially fasted for 24 h, with free access to water. After anesthesia with ketamine (100 mg/kg, i.p) and xylazine (5 mg/kg, i.p) both groups were submitted to median laparotomy. Animals in the truncal vagotomy group were submitted to the vagus nerve section through an esophageal serotomy ~1.5 cm above the cardia. On the other hand, those in the splanchnotomy + celiac gangliectomy group underwent median laparotomy with exposure of the abdominal viscera and the celiac trunk. Then, the celiac ganglion and splanchnic nerves were sectioned, followed by a 100% alcohol instillation^9^ ^,^ ^13^. After two days, the gastric emptying protocol was applied. 

### Statistical analysis

The graphs and statistical analysis of the results were performed using the Prisma^®^ software version 5.01 (GraphPad, San Diego, USA). The results of gastric emptying and intestinal transit were presented as a histogram that is representative of the ± SEM. The statistical differences between the means were analyzed with the Student’s t-test. The gastric compliance results were presented in a box chart and wisker plots. The statistical differences in the means obtained in different groups of animals in the study of the vagal and splanchnic pathways were analyzed by ANOVA followed by the Bonferroni test. p <0.05 values were considered significant.

## RESULTS

Rotations of the animal around the axis on the opposite side of the lesion, induced by apomorphine, reflect the hypersensitivity and severity of the damage caused by the intra-striatal lesions produced by the 6-OHDA injection^17^. In this study, the average contralateral rotations counted during rotational testing (60 min) of the animals in the control group was 11.17±3.38, while in the 6-OHDA group it was 358.32 ± 25.55. 

Retention of the test meal in the stomach of animals in the 6-OHDA group 10 min postprandial was 24.85% higher than in the control group, whose mean dye retention was 53.40±2.80 vs. 42.77±2.65, respectively. Dye retention in the proximal portion of the small intestine of animals in the 6-OHDA group was 18.21% lower than in the control group, whose dye retention averages were 29.19±1.95 and 35.69±1.31, respectively (Figure 2 A).

The retention of the test meal in the stomach of animals in the 6-OHDA group 20 min postprandial was 31.52% (33.50±2.53 vs. 44.06±2.23) higher than the control group. In the proximal portion of the small intestine of animals in the 6-OHDA group, there was a reduction of 32.68% (26.74±1.65 vs. 18.00±2.55) of dye retention when compared to animals in the control group (Figure 2 B).

The retention of the test meal by the stomach of animals in the 6-OHDA group 20 min postprandial was 33.04% (27.72±1.96 vs. 36.88±2.19) higher than the control group. In the proximal portion of the small intestine of animals in the 6-OHDA group, there was a 61.86% reduction (22.89±1.03 vs. 8.73±1.76) of dye retention when compared to animals in the control group (Figure 2 C).

**FIGURE 1:**
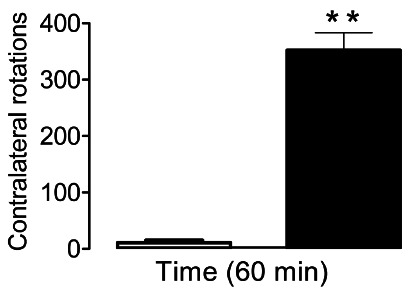
Contralateral rotations (lesion opposite side) induced by apomorphine (3 mg/kg) 13 days after surgery to induce neurodegeneration. In the graph, the white bar (□) represents the number of contralateral rotations of the animals in the control group (NaCl, 0.9%) and the black bar (■) represents the experimental group (6-OHDA). The vertical lines above each bar indicate the standard error of the mean. The animals whose contralateral rotations were >150 in 60 min were considered sensitive to the neurodegeneration induction model.

**FIGURE 2:**
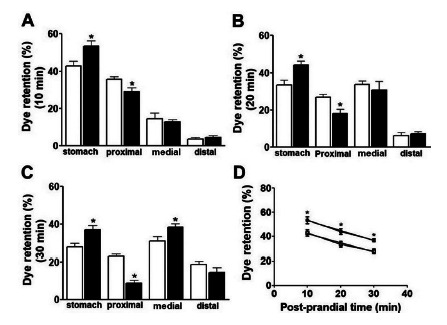
Retention of the test meal (phenol red+5% glucose) in the stomach and small intestine (proximal, meddle and distal) of the animals submitted to the neurodegeneration induction model. In the graphs, the white bars (□) represent the retention values of the test meal in each portion of the gastrointestinal tract of the animals in the control group (NaCl, 0.9%) and the black bars (■) represent the experimental group (6-OHDA). The retention of the test meal 10, 20 and 30 min postprandial are shown in Figures A, B and C respectively. Figure D studies the gastric emptying curve 10, 20 and 30 min postprandial.

**FIGURE 3:**
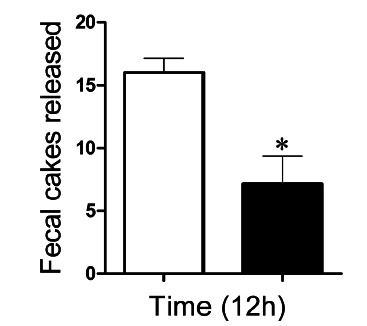
Number of fecal boluses eliminated by animals submitted to the Parkinson’s disease induction model during 12 h. The white bar (□) represents the number of fecal boluses eliminated by the animals in the control group (NaCl, 0.9%) and the black bars (■) represent the experimental group (6-OHDA).

The number of fecal boluses released by the animals in the experimental group (6-OHDA) was 6.571±1.88, while the control group (NaCl 0.9%) was 16.03±0.72 (Figure 3).

**FIGURE 4:**
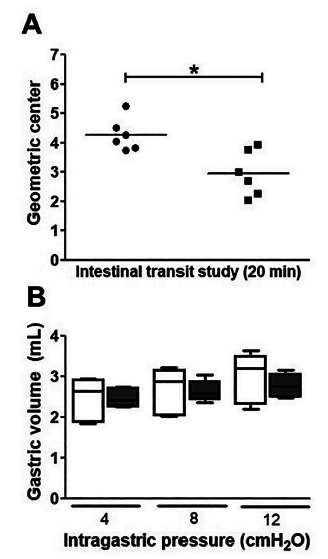
Assessment of intestinal transit and gastric compliance: A) Geometric center of the test meal 20 min postprandial, where the circles (●) represent the average retention values of the test meal in the gastrointestinal tract of animals in the control group and the squares (■) represent the experimental group (6-OHDA); B) gastric volume of animals at different intragastric pressures (4, 8 and 12 cmH_2_O). The white boxes (□) represent the animals in the control group (NaCl, 0.9%) and the black boxes (■) represent the experimental group (6-OHDA). The horizontal, lower and upper lines represent the median, lowest and highest values obtained, respectively, during 10 min of monitoring.

There was a 44.80% delay in the center of mass of the test meal in animals in the 6-OHDA group compared to those in the control group. When in animals in the 6-OHDA group, the center of mass was observed between segments 2 and 3 (2.95±0.32) of the small intestine, while in the control group (NaCl 0.9%) it was between segments 4 and 5 (4.27±0.22, Figure 4 A). 

The values of intragastric pressure in animals in the 6-OHDA and in the control group were 2.44±0.24 vs. 2.46±0.08 ml (4 cmH_2_O); 2.64±0.26 vs. 2.63±0.11 ml (8 cmH_2_O) e 2.96±0.27 vs. 2.77±0.11 ml (12 cmH_2_O), respectively. Thus, the gastric volume values of animals in the 6-OHDA and control groups did not show statistically significant differences, p <0.05 (Figure 4 B). 

**FIGURE 5:**
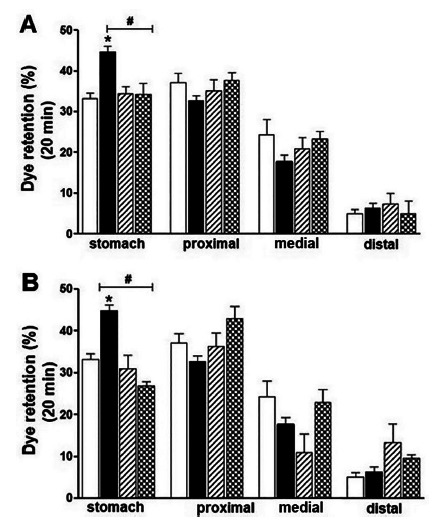
Effects of surgical pretreatments with vagotomy and splanchnotomy on gastric emptying in animals submitted to the 6-OHDA-induced neurodegeneration model: A) The white bars (□) represent the retention of the test meal in each portion of the gastrointestinal tract of the animals in the control group (NaCl, 0.9%), the black bars (■) represent the experimental group (6-OHDA), the bars with diagonal lines represent the control group pretreated with the truncal vagotomy (NaCl + VGt) and the dotted bars represent the experimental group pretreated with truncal vagotomy (6-OHDA + VGt); B) the white bars (□) represent the retention of the test meal in each portion of the gastrointestinal tract of the animals in the control group (NaCl, 0.9%), the black bars (■) represent the experimental group (6-OHDA), bars with diagonal lines represent the control group pretreated with splanchnotomy+celiac gangliectomy (NaCl, 0.9% +EGc) and the dotted bars represent the experimental group pretreated with splanchnotomy + celiac gangliectomy (6-OHDA + EGc).

Surgical pretreatment with truncal vagotomy did not alter (p>0.05) gastric fluid retention, with the results of animals in the vagotomized saline group and vagotomized 6-OHDA group (34.40±1.740 vs. 34.30±2.764) were compared to each other. Pretreatment with vagotomy accelerated gastric emptying (44.78±1.380 vs. 34.30±2.764), when the 6-OHDA group was compared with the vagotomized 6-OHDA group. On the other hand, vagotomy did not alter fluid retention in the proximal, medial and distal intestines, respectively (Figure 5A).

Surgical pretreatment with splanchnotomy + celiac ganglionectomy (EGc) did not alter (p>0.05) the results of gastric fluid retention in animals in the splanchnotomy saline and splanchnotomy 6-OHDA groups (30.94±1.740 vs. 26.30±2.764). Splanchnotomy + celiac gangliectomy accelerated gastric emptying (44.78±1.380 vs. 26.30±2.764) of the splanchnotomy 6-OHDA group in relation to the non- splanchnotomy 6-OHDA group. On the other hand, splanchnotomy+celiac gangliectomy did not significantly alter fluid retention in the proximal, medial and distal intestines, respectively (Figure 5B).

## DISCUSSION

PD was initially described as a central nervous system disease^3^. However, the idea that PD is initiated outside the central nervous system has been proposed and is being investigated. Studies show that the pathological process starts at two distinct and simultaneous points: in the olfactory bulb and in the enteric nervous system^3^. From these results, several studies based on animal models have been carried out aiming to explain the pathophysiological mechanisms of PD.

The 6-OHDA-induced neurodegeneration model is one of the most widely used. The structural similarity of 6-OHDA with dopamine and noradrenaline, allows 6-OHDA to be quickly captured by dopaminergic and noradrenergic neurons, forming lipid peroxide, as well as, the inhibition of mitochondrial complex I activity, consequently causing the death of most of these cells^14^. Therefore, in this study, the 6-OHDA PD induction model was used to expand the understanding of the pathophysiological mechanisms of PD at the gastrointestinal level. 

Contralateral rotations induced by apomorphine reflect the hypersensitivity and level of severity of striatal lesions caused by the unilateral injection of 6-OHDA^17^. In this study, the number of contralateral rotations observed during 60 min was greater than 350, thus indicating that the animals used were sensitive to the induced neurodegeneration model (Figure 1). 

The rate of gastric emptying is measured by the gastric content delivery rate in the duodenum^8^. In this study, an efficient technique, without producing ionizing radiation, based on a test meal containing a non-absorbable marker was used. The results obtained showed that PD delayed the animals’ gastric emptying in the different time periods studied (Figure 2 A, B, C). The gastric emptying curve built from the results obtained at 10, 20 and 30 min postprandial demonstrated greater retention of the test meal in the stomach of animals in the 6-OHDA group (Figure 2 D). PD caused delayed intestinal transit, evidenced from the center of mass of the test meal, when the animals in the 6-OHDA group were compared to those in the control group (Figure 4 A). In addition, a significant reduction in fecal release was also observed in animals in the 6-OHDA group, a constipation indicator, reported in approximately 90% of PD patients^12^.

These results corroborate with several studies on the effects of PD on gastric emptying in animal models. Studies carried out with rats injured with 6-hydroxydopamine reported delayed gastric emptying and constipation^20^. Neurochemical and neurophysiological changes in the intestinal-brain axis of rats with 6-OHDA-induced neurodegeneration have been reported^18^. Radiological analyzes performed on rats submitted to neurodegeneration showed delayed gastric emptying and constipation^19^. Studies carried out recently, showed that gastrointestinal motility disorders, such as delayed intestinal transit, slow fecal release and low moisture content were the first symptoms observed in the animals studied^7^.

PD did not interfere with the animals’ gastric compliance at the different pressures studied (Figure 4B). This result suggests that the delay in gastric emptying in animals with PD was caused by a delay in the opening process of the pyloric valve, consequently, retaining the test meal in the animals’ stomach for longer. This delay has important implications from the pharmacokinetic point of view, as it affects the bioavailability of levodopa, causing fluctuations in therapeutic responses, affecting the patient’s clinical condition^12^. In addition, changes in intestinal microflora have also been reported in patients with PD^10^.

The gastrointestinal tract is largely interconnected with the central nervous system and receives sympathetic and parasympathetic signs, providing extensive innervation to the plexuses of the enteric nervous system^8^. Gastric emptying is regulated by extrinsic neural pathways such as the vagal and splanchnic pathways, in addition to intrinsic innervation coordinated by the enteric nervous system^12^. Therefore, the results of this study also showed that the surgical blockage of the vagal and splanchnic routes was able to reverse the delay in gastric emptying, evidencing the involvement of such routes in the delayed gastric emptying observed (Figure 5 A, B). Recent studies have shown that the inhibitory and excitatory vagal motor circuits are responsible for the precise control of gastric emptying^8^. Therefore, in PD gastrointestinal disorders are mainly associated with an impairment of the cerebral intestinal axis, involving the efferent fibers of the vagal pathway projected directly into the gastric myenteric plexus^14^. Interestingly, blocking the brain-intestinal connection by surgically cutting the trunk branch of the vagus nerve would reduce PD disorders^16^.

PD delayed gastric emptying and intestinal transit, whose disorders can affect the bioavailability of drugs and nutrient absorption, worsening the patient’s clinical condition. The delay in gastric emptying was reversed after the surgical intervention of the truncal vagotomy and splanchnotomy and celiac gangliectomy, suggesting the involvement of such pathways in the pathophysiology of the disease. Thus, the 6-OHDA-induced neurodegeneration was presented as a model capable of mimicking the main symptoms of PD, enabling a greater understanding of the effects of this disease on gastrointestinal motility, making it possible to diagnose disorders with greater precision and leading to discoveries of pharmacological interventions for more efficient treatment.

## CONCLUSION

PD compromises gastric emptying, as well as intestinal transit, but does not alter gastric compliance. These factors together contribute to the worsening of symptoms by interfering in drug therapy absorption. The delay in gastric emptying was reversed by truncal vagotomy, splanchnotomy and celiac ganglionectomy, suggesting the involvement of such pathways in delaying gastric emptying.
